# *Toxoplasma gondii* Infection Induces Suppression in a Mouse Model of Allergic Airway Inflammation

**DOI:** 10.1371/journal.pone.0043420

**Published:** 2012-08-28

**Authors:** Ignacio M. Fenoy, Romina Chiurazzi, Vanesa R. Sánchez, Mariana A. Argenziano, Ariadna Soto, Mariano S. Picchio, Valentina Martin, Alejandra Goldman

**Affiliations:** Centro de Estudios en Salud y Medio Ambiente (CESyMA), Escuela de Ciencia y Tecnología, Universidad Nacional de San Martín, Buenos Aires, Argentina; Obihiro University of Agriculture and Veterinary Medicine, United States of America

## Abstract

Allergic asthma is an inflammatory disorder characterized by infiltration of the airway wall with inflammatory cells driven mostly by activation of Th2-lymphocytes, eosinophils and mast cells. There is a link between increased allergy and a reduction of some infections in Western countries. Epidemiological data also show that respiratory allergy is less frequent in people exposed to orofecal and foodborne microbes such as *Toxoplasma gondii*. We previously showed that both acute and chronic parasite *T. gondii* infection substantially blocked development of airway inflammation in adult BALB/c mice. Based on the high levels of IFN-γ along with the reduction of Th2 phenotype, we hypothesized that the protective effect might be related to the strong Th1 immune response elicited against the parasite. However, other mechanisms could also be implicated. The possibility that regulatory T cells inhibit allergic diseases has received growing support from both animal and human studies. Here we investigated the cellular mechanisms involved in *T. gondii* induced protection against allergy. Our results show for the first time that thoracic lymph node cells from mice sensitized during chronic *T. gondii* infection have suppressor activity. Suppression was detected both *in vitro,* on allergen specific T cell proliferation and *in vivo,* on allergic lung inflammation after adoptive transference from infected/sensitized mice to previously sensitized animals. This ability was found to be contact- independent and correlated with high levels of TGF-β and CD4^+^FoxP3^+^ cells.

## Introduction

Allergic asthma is an airway chronic inflammatory disease characterized by increased allergen specific IgE production, predominant eosinophilic airway inflammation, increased mucus secretion and development of *in vivo* hyperreactivity dependent on increased production of Th2 cytokines [1]. The phenotype expression is dependent upon the interaction between multiple factors including genetic susceptibility, infections and environmental exposures. In fact, these two last ones are the major responsible for the observed increased prevalence and morbidity of atopic disorders over the past few decades, especially in developed and developing countries [2]–[3]. This conclusion initially came from epidemiological data that allowed establishing a link between decreased childhood infections and increased allergy in western countries [4]–[6]. Later, many clinical and experimental studies with several microbes or their products supported the hypothesis [7]–[14].

It has been first proposed that the protective effect of microbial exposure might be mediated by microbe-induced Th1 cytokines such as IFN-γ [6]. Nevertheless, the immunological bases are controversial and it is increasingly clear that an imbalance between immunoregulatory and Th2 effector mechanisms can modulate allergy. It has been suggested that allergic responses are normally suppressed by regulatory cells, including CD4^+^CD25^+^ FoxP3^+^ and IL-10 Tregs (reviewed in [15]–[16]). Data from human studies showed that CD25^hi^FoxP3^+^ T-cell numbers and function were reduced in bronchoalveolar lavage samples from children with asthma compared with those seen in control subjects [17], and also that FoxP3 expression by circulating CD4^+^CD25^hi^ T cells was reduced in asthmatic patients [18]. In murine models of allergic airway inflammation many infections or products from the infectious microorganisms protect from the development of allergy by inducing the production of regulatory cytokines such as IL-10 and TGF-β [11], [19]–[22]. Hence, new approaches to allergy intervention have focused on restoring regulation (reviewed in [23], [24]). For example, Navarro *et al*. recently showed that oral treatment with bacterial extracts could prevent allergic airway disease in mice through IL-10-dependent and MyD88-dependent mechanisms and conversion of FoxP3^-^ T cells into FoxP3^+^ regulatory T cells [25].

Epidemiological studies showed that respiratory allergy is less frequent in people exposed to orofecal and foodborne microbes such as *Toxoplasma gondii* and hepatitis A virus but not to viruses transmitted through other routes [26]–[27]. Infection with *T. gondii* leads to the induction of a strong cell mediated immunity characterized by a highly polarized Th1 response in early stages of infection which is maintained during chronic infection [28]–[29]. By using a well-known murine model of allergic lung inflammation, we previously showed that both acute and chronic infection with *T. gondii* before allergic sensitization substantially blocked the development of airway inflammation in adult BALB/c mice as shown by a decrease in bronchoalveolar lavage (BAL) eosinophilia, cell infiltration around airways and vessels and goblet cell hyperplasia. Low levels of allergen-specific immunoglobulin IgE and IgG1 and high levels of allergen-specific IgG2a serum antibodies were detected. A decreased IL-4 and IL-5 production by lymph node cells was observed and a trend to an increase in IFN-γ production was detected in mice sensitized during acute infection. Noteworthy, no antigen-specific IFN-γ increase was observed when animals were sensitized during chronic infection [30]. The high levels of IFN-γ induced by the parasite along with the reduction of allergen specific Th2-associated cytokines and IgG isotypes suggested that the protective effect might be related to the high concentrations of Th1 cytokines associated with the immune response against *T. gondii*. Nevertheless, other mechanisms could also be participating, particularly when sensitizing during chronic toxoplasmosis when lower levels of IFN-γ are present in response to infection. Uncontrolled inflammatory Th1 cell responses, which eliminate intracellular pathogens such as *T. gondii*, frequently cause immunopathology so regulatory cells/cytokines control this deleterious side effect (reviewed in [31]–[32]). It is known that *T. gondii* infection elicits the production of anti-inflammatory cytokines including IL-10 and TGF-β [33] that inhibit IFN-γ production [34] and impair macrophage activation [35]. We also previously detected in chronically *T. gondii* infected mice that had been either allergen-sensitized or not, higher levels of IL-10 in BAL compared to allergic or naïve animals [30]. Characterization of the regulatory cells triggered during acute and chronic toxoplasmosis has not been completed. Conventional T-bet^+^ FoxP3^−^ Th1 lymphocytes raised in spleen and peritoneum after *i.p*. *T. gondii* infection are also capable of secreting biologically active IL-10. Also, it was recently demonstrated that CD4^+^FoxP3^+^ T cells play an important role in the modulation of the protective immune response against *T. gondii* infection during acute phase. [36]–[37].

Since the acute stage of *T. gondii* infection lasts only 10–14 days, it’s very likely that the epidemiological studies mentioned above are based on people sensitized during the chronic phase of the infection. Hence, the aim of the present study was to extend our earlier observations by investigating the cellular mechanisms involved in *T. gondii* induced protection against allergy when sensitization is carried out during the chronic phase. We found that thoracic lymph node (TLN) cells from mice sensitized during chronic *T. gondii* infection have a suppressor activity on allergen specific T cell proliferation. Suppression of effector functions, downstream of allergen sensitization, is responsible for protection from airway inflammation, as down-regulation can be transferred by TLN cells from infected/sensitized mice to uninfected, previously sensitized animals. This ability was found to be contact- independent and correlated with high levels of TGF-β and CD4^+^FoxP3^+^ cells.

## Results

### *In vitro* Suppressive Activity by Thoracic Lymph Node Cells from *T. gondii* Infected and Sensitized Mice

**Figure 1:**
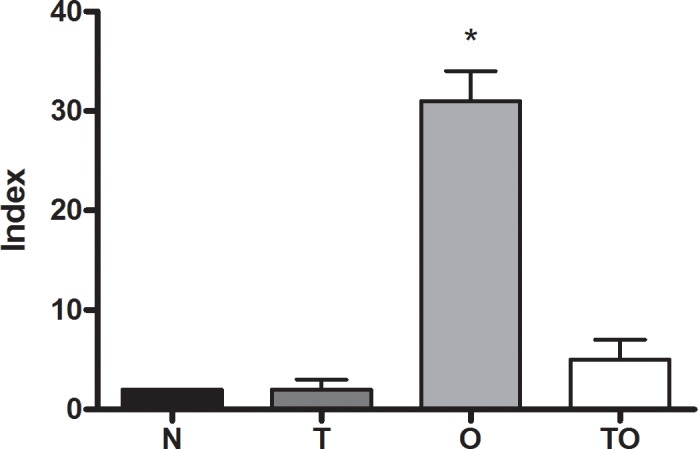
Reduced allergen specific T cell proliferation. Proliferative responses of thoracic lymph nodes cells (3×10^5^) from naïve (N), non sensitized *T. gondii* infected (T), allergic (O) and infected/sensitized (TO) mice were determined by ^3^H-thymidine incorporation after a 5-day culture period upon stimulation by OVA. Results are expressed as an Index (incorporation by cells stimulated over those cultured with medium alone). * p<0.01 vs all other groups; ANOVA with Bonferroni *a posteriori.*

Suppression of T-lymphocyte responses occurs in many infectious diseases [38]–[42] and may account for evasion of the host immune system and persistence of the microorganisms during the life of the host. Activation of various immunoregulatory cells has been demonstrated in these pathological events [31]–[32]. Also, immune regulation may ameliorate exaggerated Th1 responses against the infectious agents. With these latter in mind and to explore the cellular mechanisms involved in *T. gondii* induced protection against allergy we first evaluated if *T. gondii* infection could modulate allergen-specific T cell proliferation in BALB/c mice. Two days after allergen challenge, proliferation of TLN cells was assayed by ^3^H-thymidine incorporation during *in vitro* culture in the presence of albumin from chicken egg (OVA). OVA sensitization during chronic *T. gondii* infection (TO group) resulted in a 6.2-fold decrease in antigen-specific T cell proliferation compared to allergic mice (O). Moreover, this decrease was so remarkable that no significant differences were observed in TLN proliferation levels between the TO group and the naïve and non-sensitized *T. gondii* infected animals (T group) (Fig. 1).

**Figure 2:**
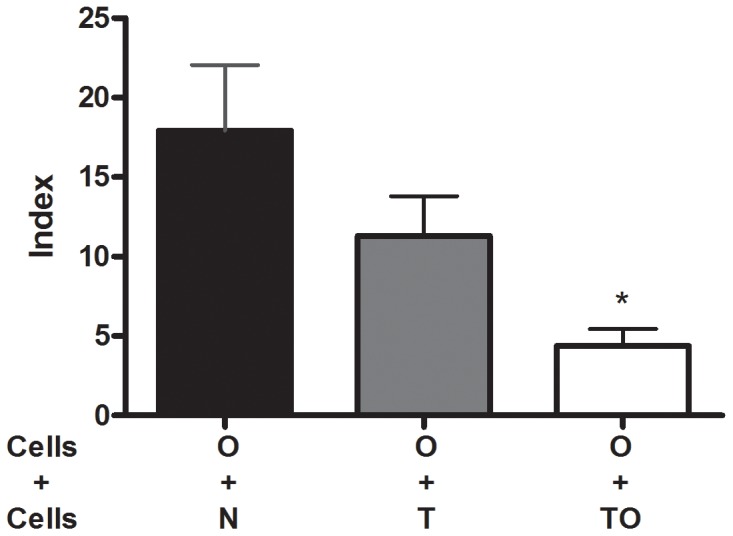
Thoracic lymph nodes cells suppressive activity. TLN cells (2.5×10^5^) from naïve (N), non sensitized *T. gondii* infected (T) and infected/sensitized (TO) mice were *in vitro* co-cultured with thoracic lymph nodes cells (2.5×10^5^) from allergic (O) mice. Proliferative responses were determined by ^3^H-thymidine incorporation after a 5-day culture period upon OVA stimulation. Results are expressed as an Index (incorporation by cells stimulated over those cultured with medium alone). * p<0.01 TO vs N; ANOVA with Bonferroni *a posteriori*.

We next studied whether the reduced proliferative response of TLN cells upon OVA stimulation could be attributed to the presence of regulatory cells. Thus, the ability of TLN cells from TO mice to suppress *in vitro* T cell proliferation against OVA was studied. TLN cells from allergic mice were co-cultured with TLN cells from TO, T, or naïve mice and stimulated with OVA. T cell proliferation from allergic mice was significantly diminished when co-cultured with TLN cells from TO mice. TLN cells from normal mice did not affect proliferation levels (Fig. 2). Interestingly, when cells from allergic mice (O group) were co-cultivated with TLN cells from non sensitized *T. gondii* infected mice (T) a trend toward decreased allergen-specific T cell proliferation was also observed. These results suggest that thoracic lymph node cells from previously infected/sensitized animals (TO) have a suppressor activity on allergen-specific T cell proliferation.

**Figure 3:**
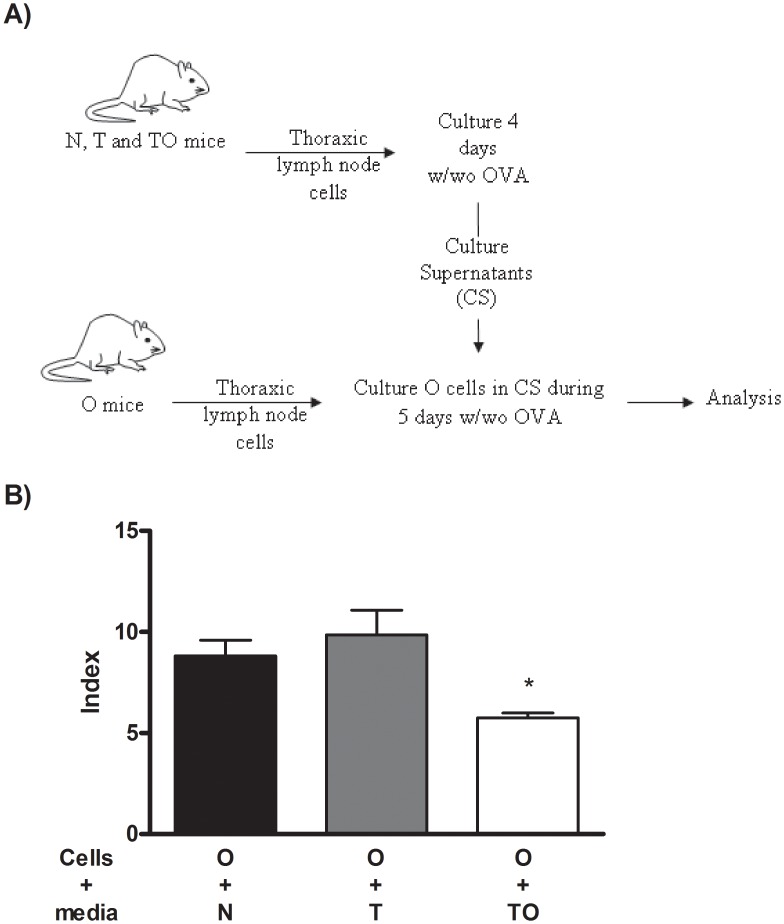
Cell supernatants suppressive activity. Thoracic lymph nodes cells (2.5×10^5^) from naïve (N), non sensitized *T. gondii* infected (T) and infected/sensitized (TO) mice were *in vitro* cultured during 4-days upon OVA stimulation. Cell supernatants were collected and used to culture thoracic lymph nodes cells (2.5×10^5^) during 5 days upon OVA stimulation. Proliferative responses were determined by ^3^H-thymidine incorporation. Results are expressed as an Index (incorporation by cells stimulated over those cultured with medium alone). * p<0.05 TO vs N and T; ANOVA with Bonferroni *a posteriori*.

To investigate whether cell-cell contact between TO and allergic TLN cells was required for the suppressor activity, cell supernatant derived from TO TLN cells previously stimulated for 4 days with OVA was added to TLN cells from allergic (O) mice. Figure 3A delineates the experimental design. After stimulation for 5 days with OVA, a reduced T cell proliferative response was detected when TLN cells from allergic mice were cultured with supernatants from TO TLN cells compared to supernatants from naïve (N) or non sensitized *T. gondii* infected TLN cells (T) (Fig. 3B).

### Production of Local Regulatory Cytokines

**Figure 4:**
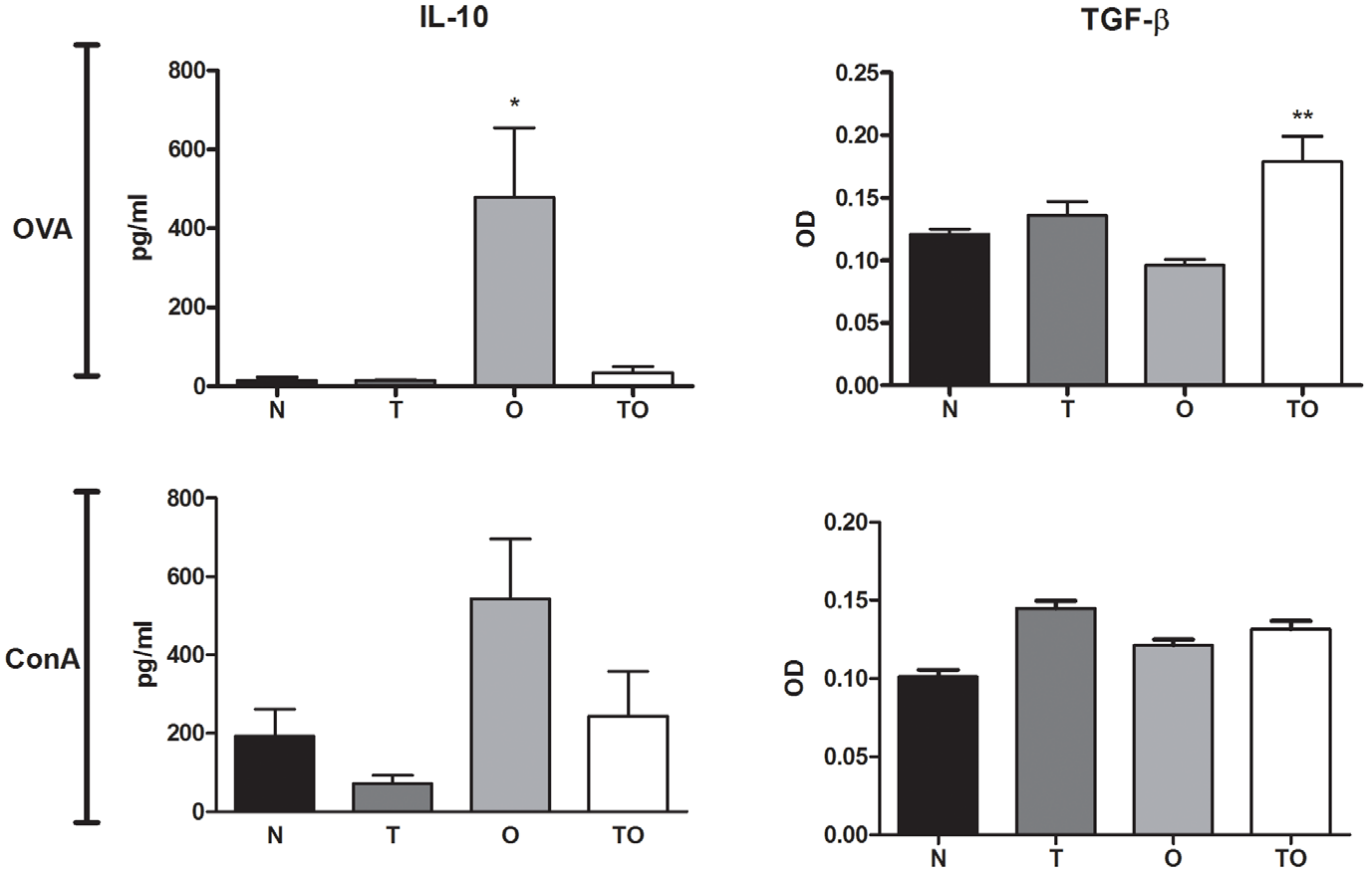
Production of local regulatory cytokines. Cytokine production by thoracic lymph node cells cultured with OVA or ConA in vitro were measured in mice OVA-sensitized (O), *T. gondii* infected/OVA sensitized (TO) (both groups aerosolized with OVA), *T. gondii* infected (T) or naïve (N) mice (both negative groups aerosolized with PBS).*p<0.05 O vs all other groups, **p<0.05 TO vs all other groups; ANOVA with Bonferroni *a posteriori*.

We previously showed that infection with *T. gondii* before OVA sensitization resulted in a decrease in Ag-specific IL-4 and IL-5 production by thoracic lymph node cells with no significant increase in IFN-γ secretion [30]. In order to study whether the suppression of allergen-specific T cell proliferation induced by supernatants from TLN cells from TO mice correlated with the presence of regulatory mediators, we analyzed IL-10 and TGF-β cytokine levels in supernatants from *in vitro* cultures. Thoracic lymph node cells from the different groups were *in vitro* stimulated with OVA or ConA. High levels of IL-10 were detected in supernatants from OVA-stimulated cells from allergic mice whereas a marked reduction was observed in mice sensitized during chronic infection (OT group). The same tendency was detected when the cells were stimulated with ConA (Fig. 4). Thus, IL-10 production behaved similarly to the Th2 cytokines already measured. TGF-β levels showed a significant increase in supernatants from chronically infected/OVA sensitized mice (TO) after *in vitro* stimulation with OVA compared to all other groups. ConA stimulation induced increased levels of this regulatory cytokine not only in the TO group but also in the non-sensitized chronically infected group (T) (Fig. 4).

### Analysis of Treg CD4^+^FoxP3^+^ Cells

**Figure 5:**
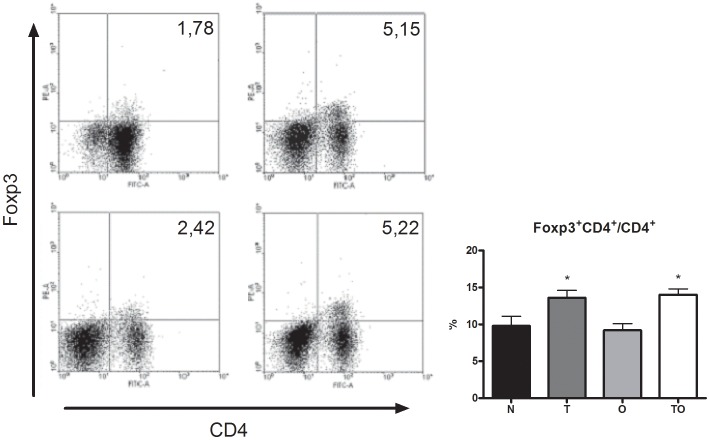
Expansion of Treg CD4^+^FoxP3^+^ cells. Flow cytometry analysis of TLN cells from naïve (N), *T. gondii* infected (T), allergic (O) and *T. gondii* infected/OVA sensitized mice (TO) stained with anti-CD4 and Foxp3. Representative dot blots from each group (A) and bar charts showing percentage of TLN cells expressing Foxp3 on CD4+ subset (B). *p<0.05 T and OT vs both N and O groups; ANOVA with Bonferroni *a posteriori*.

To assess whether the allergen-specific suppressor activity observed in thoracic lymph nodes from *T. gondii* infected/OVA sensitized group (TO) correlates with the presence of CD4^+^FoxP3^+^ cells, we evaluated the percentage of this population in all experimental groups. OVA sensitization during chronic infection resulted in a significant increase in the percentage of CD4^+^FoxP3^+^ cells, compared with allergic (O) and naïve (N) groups. Chronically infected mice (T) also showed a high percentage of this population (Fig. 5).

### Adoptive Transfer of Thoracic Lymph Node Cells from *T. gondii* Infected/Sensitized Mice Results in Protection against Allergy

**Figure 6:**
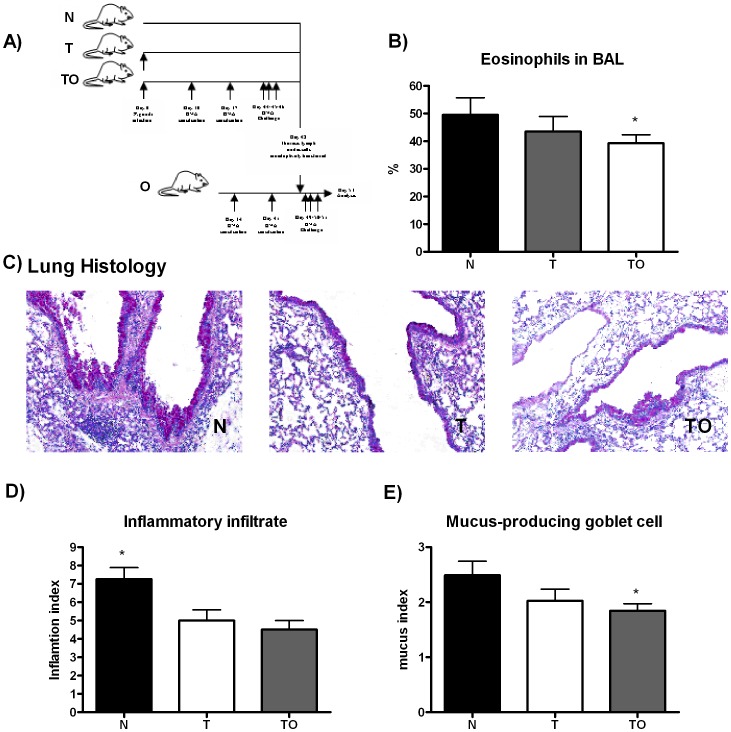
Transfer of protection against allergy with thoracic lymph node cells from *T. gondii* infected and sensitized mice. Experimental design: TLN were removed from TO, T, and naïve mice and injected iv in mice previously ip sensitized twice with OVA. Twenty four hours later, mice were exposed to aerosols of allergen on 3 consecutive days (A). Bronchoalveolar lavage was performed 48 h. after the last exposure to OVA. BAL differential cell counts were performed on cytocentrifuge slides, fixed and stained with a modified Wright-Giemsa stain. * p<0.05 TO vs N, ANOVA with Bonferroni *a posteriori* (B). After lavage, lungs were instilled and fixed with 10% buffered formalin. Following paraffin embedding, sections for microscopy were stained with Hematoxylin and PAS (C). An index of pathologic changes in H&E slides was obtained by scoring the inflammatory infiltrate around the airways and vessels for greatest severity (0, normal; 1, <4 cells diameter thick; 2, 4–10 cells diameter thick; 3, >10 cells diameter thick) and overall extent (0, normal; 1, <25% of sample; 2, 26–50%; 3, 51–75%; 4, >75%). The Index was calculated by multiplying severity by extent. * p<0.05 N vs T and TO, ANOVA with *Bonferroni a posteriori* (D). An histological goblet cell score was obtained in Periodic acid-Schiff (PAS)-stained lung sections by examining 10 to 20 consecutive airways from all groups of mice at 40x magnification and categorized according to the abundance of PAS-positive goblet (0, <5% goblet cells; 1, 5–25%; 2, 26–50%; 3, 51–75%; 4, >75%). The Index was calculated by dividing the sum of the airway scores from each lung by the number of airways examined for the histological goblet cell score. Original magnification×200. * p<0.05 N vs TO; ANOVA with Bonferroni *a posteriori* (E).

To evaluate whether thoracic lymph node cells from mice sensitized during *T. gondii* infection (TO) can suppress an allergic lung inflammation, we adoptively transferred TLN cells from this experimental group to OVA-sensitized mice. Since TLN cells from *T. gondii* infected (T) animals showed a trend to suppress an allergic T cell proliferative response, transference of TLN cells from this group was also analyzed. The cells were *iv* inoculated in mice that had received two OVA/Al(OH)3 *ip* injections. The control group included mice transferred with TLN cells from naïve mice. One day later, receptor mice were aerosol challenged with the allergen for three consecutive days (Fig. 6A). The differential count showed decreased eosinophilia in BAL from animals transferred with TO TLN cells compared with those transferred with naïve cells. A tendency to decreased eosinophilia was also detected when of TLN cells from non sensitized infected mice (T) were adoptively transferred, still not statistically different (Fig. 6B). The H&E and PAS lung-stained sections were analyzed to evaluate whether the diminished BAL eosinophilia correlated with a reduction in lung pathology. Allergic mice transferred either with TO or T TLN cells showed smaller inflammatory infiltrate with diminished goblet cell hyperplasia compared to allergic mice transferred with normal cells (Fig. 6C). The results of semiquantitative scoring of histology support the qualitative changes (Fig. 6D and E).

### IL-10 does not Appear to Play a Role in *T. gondii*-induced Protection against Allergy

**Figure 7:**
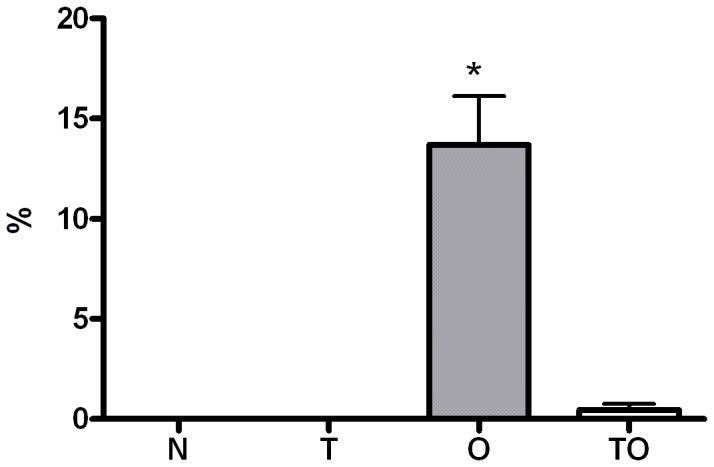
Diminished eosinophilia in IL-10 KO mice bronchoalveolar lavage (BAL). IL-10 KO mice were OVA sensitized during chronic *T. gondii* infection. BAL was performed 48 h after the last exposure to OVA. Differential cell counts were performed on cytocentrifuge slides, fixed and stained with a modified Wright–Giemsa stain. * p<0.05 versus all other groups; ANOVA with Bonferroni *a posteriori*.

IL-10 overexpression was shown to be an important mediator of allergic protection in different experimental models of infections or treatments with microbial products [22], [25], [43], [44]. Though the results obtained herein when supernatants from lymph node cells stimulated with OVA were analyzed for IL-10 production do not suggest that this cytokine could be involved in allergic protection, we had previously showed that higher levels of this regulatory cytokine were found in bronchoalveolar lavage from infected mice compared to normal and allergic animals [30]. Therefore, IL-10-deficient mice were employed to confirm whether (or not) IL-10 was mediating the protection from allergic inflammation when mice are sensitized during chronic *T. gondii* infection. In accordance to previous studies, which showed that IL-10 would play an important role in regulating the strong Th1 response elicited by *T. gondii* [33], IL-10-deficient mice showed increased mortality during the acute phase of infection (data not shown). Surviving animals were sensitized during the chronic phase and examined for pulmonary inflammation after OVA airway challenge. Similar results to those seen in wild type mice were obtained. Increased BAL eosinophilia and mononuclear cell infiltration around airway and vessels and mostly goblet cell hyperplasia was observed in OVA sensitized and challenged animals compared to naïve and *T. gondii* infected mice (Fig. 7). Infection with *T. gondii* before allergic sensitization resulted in a strong reduction of airway eosinophilia. BAL cells differential staining showed that eosinophils percentage was significantly diminished in *T. gondii* infected/OVA sensitized (TO) mice (Fig. 7). Also, a lower level of inflammatory infiltrate, although not significant, and a statistically significant diminished goblet cell hyperplasia compared with the O group were detected (Table 1). As can be observed the TO group shows inflammation and mucus indexes significantly lower than the allergic mice.

**Table 1:** Reduced allergic lung inflammation in IL-10 KO mice.

Group	Inflammation index	Mucus index
N	0.62±0.14	0.00±0.00
T	0.45±0.05	0.14±0.00
O	1.35±0.24*	1.08±0.27**
TO	0.98±0.19	0.54±0.19

Semiquantitative analysis of histopathological changes. After lavage, lungs were instilled and fixed with 10% buffered formalin. Following paraffin embedding, sections for microscopy were stained with H&E and PAS. An index of pathologic changes in H&E slides was obtained by scoring the inflammatory infiltrate around the airways and vessels for greatest severity (0, normal; 1, <4 cells diameter thick; 2, 4–10 cells diameter thick; 3, >10 cells diameter thick) and overall extent (0, normal; 1, <25% of sample; 2, 26–50%; 3, 51–75%; 4, >75%). The Index was calculated by multiplying severity by extent. An histological goblet cell score was obtained in Periodic acid-Schiff (PAS)-stained lung sections by examining 10 to 20 consecutive airways from all groups of mice at 40x magnification and categorized according to the abundance of PAS-positive goblet (0, <5% goblet cells; 1, 5–25%; 2, 26–50%; 3, 51–75%; 4, >75%). The Index was calculated by dividing the sum of the airway scores from each lung by the number of airways examined for the histological goblet cell score. MEAN±ES;

* p<0.05 O vs N and T groups;

** p<0.001 O vs all other groups; ANOVA with Bonferroni *a posteriori.*

These results would suggest that *T. gondii*-induced protection from allergy is not mediated by IL-10 when sensitization occurs during chronic infection.

## Discussion

Allergic asthma is an inflammatory disorder principally involving the conducting airways and characterized by infiltration of the airway wall with inflammatory cells driven mostly by activation of Th2-lymphocytes, eosinophils and mast cells. As Th1 and Th2 cell responses are mutually opposed, it has been proposed that decreased microbial stimulation of Th1 cell responses in developed or developing countries has led to allergic Th2 cell reactions. Based on epidemiological studies that showed that respiratory allergy is less frequent in people exposed to orofecal and foodborne microbes such as *Toxoplasma gondii* [26]–[27] and bearing in mind that infection with the parasite leads to the induction of a strong cell-mediated immunity characterized by a highly polarized Th1 response, we previously used a well-known mouse model of allergen sensitization and airway allergen challenges to analyze the ability of *T. gondii* to modulate allergy. We demonstrated that both acute and chronic *T. gondii* infection can block the development of allergic airway inflammation [30]. In agreement with our results, Wagner *et al*. showed that *T. gondii* infection prior to sensitization with Bet v 1 showed neither IL-5 nor eosinophils in BAL fluids and reduced Bet v 1-specific IgE antibodies along with elevated levels of rBet v 1-specific IgG2a [45]. The results presented in the present work extend those earlier studies that demonstrate a reduced allergic lung inflammation in mice infected with this parasite.

The high levels of IFN-γ secreted in response to *T. gondii* infection during the acute phase [29]–[30], let us hypothesize that the protective effect could be related to the elevated concentrations of Th1 cytokines. However, during the chronic phase, IFN-γ levels diminish [29]–[30]. Indeed, lower levels of IFN-γ were previously detected in supernatants of splenocytes stimulated with TgAg. In addition, non-stimulated splenocytes only showed significant IFN-γ levels in supernatants from acute infected mice but not in the chronic stage [30]. Still, the lower levels of this cytokine could be enough to inhibit the differentiation of Th precursors into OVA-specific Th2 lymphocytes without driving a shift towards a Th1 cellular response. Indeed, a decreased IL-4 and IL-5 production by lymph node cells and no antigen-specific IFN-γ increase was observed when animals were sensitized during chronic infection [30]. Even so, other mechanisms could also be involved. The possibility that regulatory T cells reduce allergic diseases has received increasing sustain from animal [19]–[21], [24] and human studies [17]–[18], [46]–[47]. On the other hand, it has been demonstrated that Treg cells can regulate the uncontrolled inflammatory Th1 cell immune response against infectious agents including parasites, which frequently cause immunopathology [31]–[32]. For instance, during the acute phase of diverse infections (*P. berghei, Mycobacterium tuberculosis, Plasmodium yoelii, Brugia malayi*, and *L. sigmodontis*), an increase in FoxP3^+^ cell number is observed [48]–[52]. *In vivo* depletion of CD25^+^ T cells was shown to induce an increase in IFN-γ production in mice infected with *Plasmodium chabaudi adami* [53] and *Trypanosoma congolense* [54].

Immune regulation against *T. gondii* infection is not entirely understood and characterization of the regulatory cells/cytokines triggered during acute and chronic toxoplasmosis has not been completed. It was recently demonstrated that CD4^+^FoxP3^+^ T cells play an important role in the modulation of the protective immune response against *T. gondii* infection during the acute phase [36]–[37]. Depletion of Treg FoxP3+ cells in orally infected BALB/c mice led to an increase in parasite burden, higher production of pro-inflammatory cytokines and increased ileal histopathology [37].

As mentioned in the introduction section, it is known that *T. gondii* elicits the production of anti-inflammatory cytokines such as IL-10 and TGF-β [33] that inhibit IFN-γ production [34] and impair macrophage activation [35]. TGF-β secreted by infected macrophages down-regulates TNF-α receptor expression and allows the intracellular survival of *T. gondii* (reviewed in [55]). Buzoni-Gatel *et al*. demonstrated that TGF-β is necessary for the immune regulation at the gut level. Inhibition of this cytokine results in the development of ileitis in resistant mice, such as CBA/J [reviewed in 55]. Also, splenocytes TGF-β mRNA expression levels were markedly increased during the chronic stage of infection (day 49) [45]. Voisin *et al*. [56] described the presence of a population of GR1^+^ CD11b^+^ myeloid cells with nitric oxide-dependent regulatory properties able to secrete IL-10 during acute infection. Conventional T-bet^+^ FoxP3^+^ Th1 lymphocytes raised in spleen and peritoneum after i.p. *T. gondii* infection are also capable of secreting biologically active IL-10. Moreover, studies using IL-10-deficient mice showed increased mortality during the acute phase of infection, confirming the important role of simultaneous induction of regulatory cytokines [33].

The results obtained in the present work allow to hypothesize that part of the mechanisms involved in *T. gondii* down-regulation of allergic lung inflammation might be mediated by regulatory cells. To address this, we first analyzed whether *T. gondii* infection could modulate allergen-specific T cell proliferation. *In vitro* proliferation against OVA was diminished in thoracic lymph node cells from mice sensitized during chronic *T. gondii* infection (TO) compared to allergic animals (O). The unresponsiveness of lymphocytes suggested the presence of regulatory cells in thoracic lymph nodes. Co-culture experiments showed that TLN cells from TO mice had suppressive activity on *in vitro* T cell proliferation against OVA. Interestingly, TLN cells from *T. gondii* infected mice (T) showed a tendency to suppress allergen specific T cell proliferation. Suppression mediated by TLN cells was cell contact independent since cell supernatant from TO TLN cells *in vitro* stimulated with OVA was able to inhibit allergen specific proliferation of TLN cells from allergic mice. Nevertheless and notable, the level of suppression induced by the cell supernatant was less strong than the one detected in co-cultures (1.5 vs 4.0 fold decreased).

To demonstrate that down-regulation is mediated by regulatory cells, we used an adoptive transfer system of TLN cells from infected/sensitized (TO) mice into OVA sensitized, but uninfected hosts. *In vivo* allergy suppression correlated with the suppression of proliferation in response to allergen detected *in vitro*. Adoptive transference of TLN cells from infected/sensitized (TO group) mice reduced lung inflammation. This effect was also observed when the adoptive transference was performed with non sensitized *T. gondii* infected TLN cells. These results let us hypothesize that TLN cells from not only TO group but also from T group (non sensitized *T. gondii* infected mice) have regulatory cells able to suppress *in vitro* allergen-specific T cell proliferation and also allergic inflammation *in vivo*. The ability of lymph node cells or splenocytes from mice infected with different infectious agents to suppress allergic lung inflammation was previously shown [20], [56], [57]. For example, adoptive transference of mesenteric LN cells from chronically *Heligmosomoides polygyrus* infected mice induced declines in eosinophil lung infiltration after allergen challenges [20]. In this sense different studies agree that although the different types of Treg cells are antigen-specific, they all exert suppressive activity in a non antigen-specific way (reviewed in [46]).

The cells transferred from mice sensitized during *T. gondii* infection (TO group) secreted elevated levels of TGF-β, one of the two principal T reg cell–associated down-regulatory cytokines-. Increased levels of this cytokine were detected when TLN cells were stimulated *in vitro* with OVA. *In vitro* ConA stimulation induced augmented secretion levels of TGF-β not only in the TO group but also in TLN cells from non sensitized chronic infected (T) experimental group. As previously described [30], OVA-specific IL-10 levels in TLN are diminished in TO mice compared to allergic animals. However, a significant increase in BAL was previously observed in both infected groups (TO and T) compared to allergic mice [30]. This result suggested that this cytokine could be involved in the allergy protection induced by the parasite. The experiments with IL-10 KO mice showed that the regulatory cytokine IL-10 would not play a significant role in *T. gondii* modulation since diminished BAL eosinophilia and goblet cell hyperplasia was detected in OVA sensitized infected animals (TO) compared to allergic mice. In contrast, TGF-β remains a strong candidate for immune suppression by Treg cells from *T. gondii*-infected mice, since this cytokine has the ability to induce peripheral T cells to develop regulatory competence (reviewed in [24], [58]) and it was previously reported to improve experimental airway allergy [59]–[60].

A number of different phenotypes in the regulatory T cell population involved in allergy suppression have been postulated: “Tr1” cells, able of suppressing airway allergy through the action of IL-10; “Th3” cells, mainly acting through TGF-β (also secreting low levels of IL-10) and TGF-β dependent, which were shown to play a regulatory role at mucosal sites [58]; and FoxP3^+^ T cells which, though multiple mechanisms of action have been shown *in vitro*, it is unclear whether the same or different mechanisms are used *in vivo* (reviewed in [61]). Which is the regulatory population involved in the allergy protection induced by *T. gondii*? Description of the regulatory cells elicited during acute and chronic toxoplasmosis is not fully described. As we pointed out above, *i.p. T. gondii* infection induced, in spleen and peritoneum, conventional T-bet^+^ FoxP3^+^ Th1 lymphocytes able to secrete biologically active IL-10. Also, FoxP3^+^ Tregs are important in the acute phase of *T. gondii* infection by playing an essential role in the modulation of the protective immune response against the parasite [36]–[37]. Pro-inflammatory cytokines and lamina propria parasite burden were increased in Treg cells depleted BALB/c mice [37]. In the present work, analysis of CD4^+^FoxP3^+^ cells in thoracic lymph nodes showed that *T. gondii* infection induced an expansion of this population. The percentage of CD4^+^FoxP3^+^ T cells was significantly increased in chronically *T. gondii* infected OVA-sensitized mice (TO group). Non sensitized chronic infected animals (T group) also showed a raise in this population. Wagner *et al*. detected increased number of this subset compared to naïve mice, but in mesenteric lymph nodes [45]. Hence, to our knowledge, this is the first work that demonstrates that chronic *T. gondii* infection induces an expansion in CD4^+^FoxP3^+^ T cells in lung draining lymph nodes.

Given that TLN cells from non-sensitized *T. gondii* infected animals (T group) showed to reduce lung inflammation after *in vivo* transference and also showed a tendency of suppress antigen-specific *in vitro* T cell proliferation, the fact that supernatant from this group didn’t suppress allergen-specific T cell proliferation was striking. Therefore, the mechanisms of suppression of both experimental groups, non sensitized *T. gondii* infected mice (T) and OVA sensitized chronically infected animals (TO), appear to be different. First, suppression mediated by TLN cells from the T group appears to be cell contact dependent. Maybe via FoxP3+ iTregs? Second, suppression induced by TO TLN cells is much stronger than that induced by TLN cells from non sensitized infected animals (T group). Hence, it could be hypothesized that two regulatory cell populations may be involved in the suppression of allergic lung inflammation by TO TLN cells. In addition to the regulatory cells present in non-sensitized chronically infected mice (T), another subset could be induced when chronically infected mice are OVA sensitized. This population might include Th3 cells, acting through TGF-β and cell contact independently [58] or FoxP3^+^ iTregs cells acting through an unknown soluble suppressor cytokine (reviewed in [61]). Both populations would be induced when mice are exposed to OVA. However, based on what most studies show about the cell contact dependence of Tregs FoxP3^+^ (reviewed in [15]) and that no differences in the percentage of that population was observed between TO and T animals, the last population is less plausible. It has been shown that TGF-β might be required for the development of Th3 and FoxP3^+^ iTregs cells (reviewed in [61]). Close to the time point of OVA sensitization and compared to naïve mice, increased TGF-β mRNA expression levels were detected in *T. gondii* infected mice splenocytes [45] and lung (data not shown).

Although a causal relationship between increased TGF-β production in LN cells from animals sensitized during chronic infection leading to suppression of the asthmatic phenotype was not demonstrated directly, it may be suggested that the increase of TGF-β and/or FoxP3^+^CD4^+^/Th3 cells are related to the suppressive effects observed in TLN cells from previously infected OVA sensitized mice (TO).

Understanding the mechanisms by which *T. gondii* regulate inflammation may potentially lead to the development of strategies aimed at controlling unwanted inflammation in allergic diseases. Future studies to examine the use of inactivated parasites, and particularly the identification of effective *T. gondii* component(s) should be undertaken to explore *T. gondii* as a potential immuno-modulator for the treatment of human allergic asthma.

## Materials and Methods

### Animals

BALB/c (H-2^d^) mice were obtained from the animal facilities of the ILEX-CONICET, IIHEMA, Academia Nacional de Medicina (Buenos Aires) and maintained in our animal facilities for use throughout these experiments. IL-10^−/−^ mice (BALB/c) were bred and housed at the animal facilities of Biotechnology Research Institute (IIB), National University of General San Martin (UNSAM), Buenos Aires, Argentina, and were kindly provided by J.E. Ugalde (IIB-UNSAM). Mice were used at the age of 6 to 8 weeks. All procedures requiring animals were performed in agreement with institutional guidelines and were approved by the Independent Ethics Committee for the Care and Use of Experimental Animals of National University of General San Martin (C.I.C.U.A.E., IIB-UNSAM), and approved and conducted in accordance with the guidelines established by the National University of General San Martin and the National Research Agency (PICT 562).

### Infection, Sensitization and Exposure

The Beverley strain of *T. gondii* was used in this study. For infection, cysts were obtained from the brains of orally infected C3H/HeN mice and maintained by monthly passage. BALB/c mice were orally infected with 15 cysts. One month later, sensitization was achieved by two *i.p*. injections of 0,2 ml PBS containing chicken egg white albumin (grade III Sigma-Aldrich) (20 µg) and alum (2 mg) one week apart. One week later, mice were exposed to aerosols of allergen (3% (w/v)) OVA in PBS for 10 min on 3 consecutive days (TO group). Aerosol exposure was performed within individual compartments of a mouse pie chamber using a nebulizer (SAN-UP, Argentina, OVA solution flux 0,33 ml/min in air flux of 6–8 L/min). Mice were analyzed 48 h after the last exposure. Negative controls include *T. gondii* infected (T) and non infected (naïve) mice both inoculated with alum alone and aerosolized with PBS.

### Pathologic Analysis

Animals were euthanized with sodium pentobarbital. The chest wall was opened and the animals were exsanguinated by cardiac puncture. The trachea was cannulated after blood collection. Bronchoalveolar lavage (BAL) was performed four times with 1 ml of sterile PBS instilled and gently harvested. Lavage fluid was collected, centrifuged at 300 g for 10 min, and the pellet was resuspended in 0.5 ml PBS. Total cell yield was quantified and BAL differential cell counts were performed on cytocentrifuge slides prepared by centrifugation of samples at 800 rpm for 5 min (Cytospin 4; Shandon, Pittsburg, PA). These slides were fixed and stained with a modified Wright-Giemsa stain (Tinción 15, Biopur SRL, Rosario, Argentina), and a total of 200 cells were counted for each sample by microscopy. Macrophages, lymphocytes, neutrophils, and eosinophils were quantified. After lavage the lungs were instilled with 10% buffered formalin, removed and fixed in the same solution. Following paraffin embedding, sections for microscopy were stained with H&E and PAS. An index of pathologic changes in H&E slides was obtained by scoring the inflammatory infiltrates around the airways and vessels for greatest severity (0, normal; 1, <4 cells diameter thick; 2, 4–10 cells diameter thick; 3, >10 cells diameter thick) and overall extent (0, normal; 1, <25% of sample; 2, 26–50%; 3, 51–75%; 4, >75%). The index was calculated by multiplying severity by extent [30]. An histological goblet cell score was obtained in Periodic acid-Schiff (PAS)-stained lung sections by examining 10 to 20 consecutive airways from all groups of mice at 40x magnification and categorized according to the abundance of PAS-positive goblet (0, <5% goblet cells; 1, 5–25%; 2, 26–50%; 3, 51–75%; 4, >75%)). The index was calculated by dividing the sum of the airway scores from each lung by the number of airways examined for the histological goblet cell score [30].

### Proliferation Assays and Cytokine Production

Thoracic lymph nodes (TLN) removed. Single cell suspensions were made using a cell strainer and 3×10^5^ cells were cultured in 200 µl of medium RPMI 1640 supplemented with 20% FBS (GIBCO), 1% antibiotics and 5×10^−5^ M 2-mercaptoethanol alone or in the presence of OVA (200 µg/ml) or concanavalin A (ConA) (5 µg/ml). Cytokine production was measured in supernatants at 72 h by capture ELISA commercial kits (Pharmingen, BD Biosciences for IL-10 and eBiosience for TGF-β ). ConA stimulated supernatants were harvested at 48 h. Proliferative responses of TLN cells cultured with medium or OVA (concentrations as above) were determined after addition of methyl-^3^H-thymidine (1 µCi/well, PerkinElmer, Argentina) for the last 18 h of a 5 days culture period. To evaluate *in vitro* suppression activity, TLN cells from allergic (2.5×10^5^/well) mice were co-cultured with TLN cells from TO, T, or naïve (2.5×10^5^/well) mice and stimulated with OVA or medium. To obtain cell supernatant (CS), TO, T and naïve TLN cells (2.5×10^5^) were stimulated for 4 days with OVA or medium. This CS was next added to TLN cells from allergic mice (2.5×10^5^), stimulated with OVA (200 µg/ml) and cultured for 5 days. Incorporated radioactivity was measured in a liquid scintillation beta-counter (Beckman). From the row data obtained, an index was calculated as cpm incorporated by stimulated cells over those cultured with medium alone (*e.g*: the results showed in Figure 1 were calculated from the following raw data N: 539±60 vs 261±19; T group: 731±199 vs 341±51; TO: 2448±1806 vs 374±110; O: 28810±12172 vs 888±334; mean cpm±SE, stimulated vs. non-stimulated).

### Adoptive Transfer Experiments

TLN were removed from TO, T, and naïve mice. Single-cell suspensions in RPMI–3% FBS were made using a cell strainer. 5×10^6^ cells were injected i.v. in PBS. Mice received TLN cells 7 d after a second OVA ip sensitization. Twenty four h later, mice were exposed to aerosols of allergen (3% (w/v)) OVA in PBS for 10 min on 3 consecutive days.

### Flow Cytometry Analysis

Intracellular staining of Foxp3 was performed using phycoerythrin-conjugated anti-Foxp3 mAb and the Foxp3 staining buffer set (Pharmingen, BD Biosciences) according to the manufacturer’s protocol. Fluorescein isothiocyanate-conjugated anti-CD4 mAb (clone RM4-5) from Pharmingen, BD was used. Cells were acquired on a FACScan cytometer (Becton Dickinson, Mountain View, CA). Data were analyzed by using WinMDI software.

### Statistical Analysis

Each experimental group had at least four mice and each experiment was repeated at least 4 times. Data are presented as mean ± SEM. Statistical analysis was performed using ANOVA analysis of differences among groups with Bonferroni test “*a posteriori*” as indicated in the figure legends. Statistical significance was accepted when p<0.05.
